# Fat free mass index and fat mass index tracing on body composition chart in diabetes adolescent girls

**DOI:** 10.1186/1687-9856-2015-S1-P20

**Published:** 2015-04-28

**Authors:** Sochung Chung, Hye Won Park, Byung Ok Kwak, Kyo Sun Kim

**Affiliations:** 1 Department of Pediatrics, Konkuk University Medical Center, Seoul, Korea; 2 Konkuk University School of Medicine, Seoul, Korea

**Keywords:** Body composition, Diabetes mellitus, Adolescent

## Aims

The prevalence of obesity and type 2 diabetes is increasing, and the impact of obesity on diabetes manifestation should be considered in management of type 1 diabetes children. There are some cases difficult to categorize into certain type in pediatric diabetic patients in the era of acceleration. The objective of this study was to determine the type differences of diabetes by analyzing the growth and body composition status and choose a proper treatment modality using the body composition chart.

## Methods

Fourteen type 1 diabetic adolescent girls (age 12.6±3.3 years) and 16 type 2 diabetic adolescent girls (age 14.2±2.6 years) were included. Height, weight and body compartment of fat mass, fat free mass were measured in each patient. Body mass index (BMI), fat mass index (FMI), fat free mass index (FFMI) and percent body fat (PBF) were calculated. FFMI and FMI were plotted on body composition chart and traced the coordinate during follow up period.

## Results

BMI difference between diabetes types was explained with the difference in FFMI as well as FMI. Body composition chart presented that type 2 diabetes girls showed marked elevation in FMI and PBF. And FFMI was lower in type 1 diabetes girls. Superimposed effect of obesity on type 1 diabetes was expressed on body composition chart with marked increment of FMI and the adequate effect of lifestyle intervention in type 2 diabetes was showed marked decrease in FMI and sustained FFMI during follow up period.

## Conclusion

Body composition chart might be useful in adequate growth and glucose control monitoring in diabetes children and adolescents. Early adjustment of diabetes management strategy on the basis of body composition during growing period will improve glucose control and promote adequate growth.

